# Development of a real-time quantitative PCR method for detection and quantification of *Prevotella copri*

**DOI:** 10.1186/s12866-020-02063-4

**Published:** 2021-01-11

**Authors:** Phebe Verbrugghe, Olivier Van Aken, Frida Hållenius, Anne Nilsson

**Affiliations:** 1 Food Technology, Engineering and Nutrition, Lund University, PO Box 124, 221 00 Lund, Sweden; 2 Department of Biology, Lund University, Lund University Plant Sciences, Sölvegatan 35, 223 62 Lund, Sweden

**Keywords:** *Prevotella copri*, qPCR, SYBR green

## Abstract

**Background:**

Since its discovery in 2007, the importance of the human gut bacterium *Prevotella copri* (*P. copri*) has been widely recognized with its links to diet and health status and potential as next generation probiotic. Therefore, precise, convenient and cost-effective diagnostic tools for the detection and quantification of *P. copri* from clinical and environmental samples are needed.

**Results:**

In this study, a Sybr Green qPCR protocol for *P. copri* detection and quantification was developed and tested on *P. copri*-spiked murine faeces samples targeting both the 16S rRNA gene and *P. copri* genome specific genes. The use of one 16S rRNA primer pair and 2 genome specific primer pairs resulted in at least 10x higher specificity and sensitivity than the primer-only PCR currently cited in the literature, reaching a sensitivity of 10^3^ CFU/mL. Furthermore, we showed that the new 16S rRNA primer set provided the best balance of detection of a wide range of *P. copri* strains, while avoiding off-target detection of other *Prevotella* genus species. The quantification of *P. copri* in human stool samples using the new 16S rRNA primers also correlated well with 16S rRNA high throughput MiSeq sequencing data (*r*^2^ = 0.6604, *p* = 0.0074). The two genome specific primer pairs on the other hand uniquely detect the DSM18205 reference strain, allowing differential detection of indigenous and experimentally administered *P. copri* populations. Finally, it was shown that SYBR green qPCR mixes have an influence on sensitivity and specificity, with Biorad SsoAdvanced Universal SYBR Green Supermix performing the best under our test conditions of six commercially available SYBR green master mixes.

**Conclusions:**

This improved qPCR-based method will allow accurate *P. copri* identification and quantification. Moreover, this methodology can also be applied to identify other bacterial species in complex samples.

## Background

Human microbiome research has grown exponentially over the last 15 years [1] and many studies have shown an increase or decrease of certain bacterial species in various disease conditions [2]. These reports imply the use of specific bacterial species as potential biomarkers for diet and disease, or as prevention or therapy of diseases. One common human gut bacterial species that has been associated with diet and disease is *Prevotella copri* (*P. copri*). These obligate anaerobic gram-negative rods were first isolated from the human faeces of a Japanese man [3]. They are common in the human gut of communities in under-developed/non-westernised countries [4–6], and their abundance has been linked to healthy vegetable and fiber-rich diets [5]. Regarding its links to health status, decreased levels of *P. copri* were observed in obese women [7] and in subjects with neurodegenerative diseases [8] and childhood atopic dermatitis [9]. In healthy human subjects, increased *P. copri* abundance was correlated with improved glucose tolerance after barley consumption; and in mice, oral administration of live *P. copri* resulted in improved glucose metabolism [10], suggesting a causal relationship. In line with these findings, *P. copri* has been identified as a candidate for a next generation probiotic to prevent and treat metabolic diseases [11]. On the other hand, increased levels of *P. copri* were reported in patients with new onset untreated rheumatoid arthritis [12] and Irritable Bowel Syndrome [13]. Considering faecal *P. copri* has been associated with both health benefits and some inflammatory states, it is important to have accurate and rapid methods for testing its abundance.

Until recently, identification of bacterial species from the human gut relied on culture-dependent methods. This lead to a substantial underestimation of biodiversity as the human gut contains bacteria that are either slow growing or unculturable that cannot be detected in this way [14]. The introduction of genetic markers such as the 16S rRNA gene, and the development of extensive bacterial genome databases have lessened the need for bacterial cultures, with real-time quantitative PCR (qPCR) now widely used as a technique to identify and quantify target species in a microbial community sample. The 16S rRNA gene is present in all bacteria and contains both conserved and hypervariable regions. Universal primers can be designed in the conserved regions while the interspersed hypervariable regions enable discrimination between bacterial families, genera and species.

In our hands, the primer-only PCR currently cited in the literature to detect *P. copri* targeting FimB/Mfa2 family fimbrial subunit (PREVCOP_RS00015) [12] is not sensitive in murine faeces. Indeed, complex biological samples such as faeces and gut content extracts have been shown to complicate accurate analysis [15]. PCR primers and a Taqman probe specific to the V3 region of *P. copri* were recently described by Gray et al. [16] but here we sought to develop a solely primer-based cost effective Sybr Green method.

In this study, 16S rRNA and species-specific primers have been developed to detect and quantify *P. copri* in murine and human faeces, achieving improved *P. copri* quantitative real-time PCR specificity and sensitivity compared to the primers most commonly cited in the literature [12].

## Results

### *P. copri*-specific primer-only PCR currently cited in the literature lacks specificity in complex biological samples

**Fig. 1:**
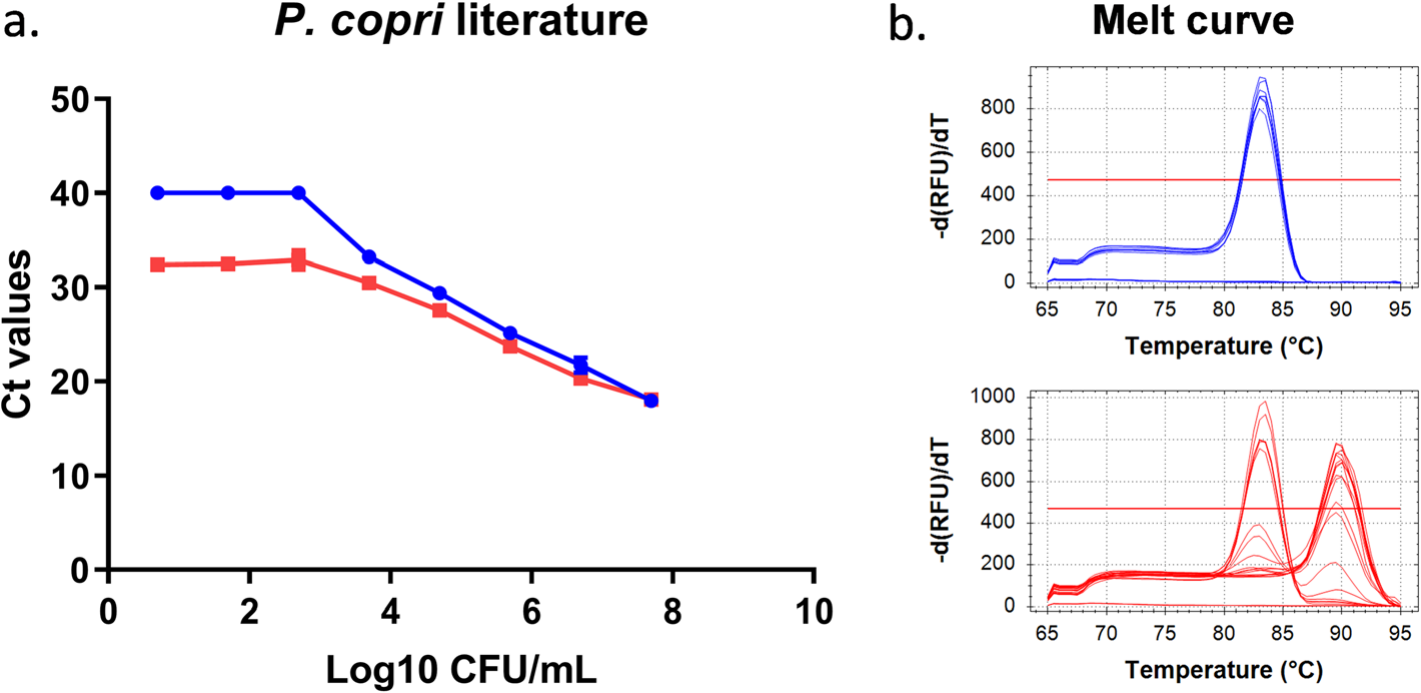
Performance of *P. copri* genome specific primers from the literature [12] under stringent conditions. Standard curves and melt curves for *P. copri* only (blue) and faeces spiked with *P. copri* (red)

The primers most commonly used in the literature to detect *P. copri* [12] targeting FimB/Mfa2 family fimbrial subunit (PREVCOP_RS00015) were initially tested. Though the primers showed good specificity and sensitivity for pure *P. copri* gDNA samples, this was not the case in the presence of murine faeces, where non-specific amplification was observed in the melt curves (multiple peaks indicating different products) at low *P. copri* concentrations even under stringent PCR conditions (Fig. 1). The standard curves show that the reliable detection threshold is lower when no faeces are present (10^4^ CFU/mL versus 10^5^ CFU/mL when faeces are present).

### New *P. copri* 16S rRNA primers improve specificity

**Fig. 2:**
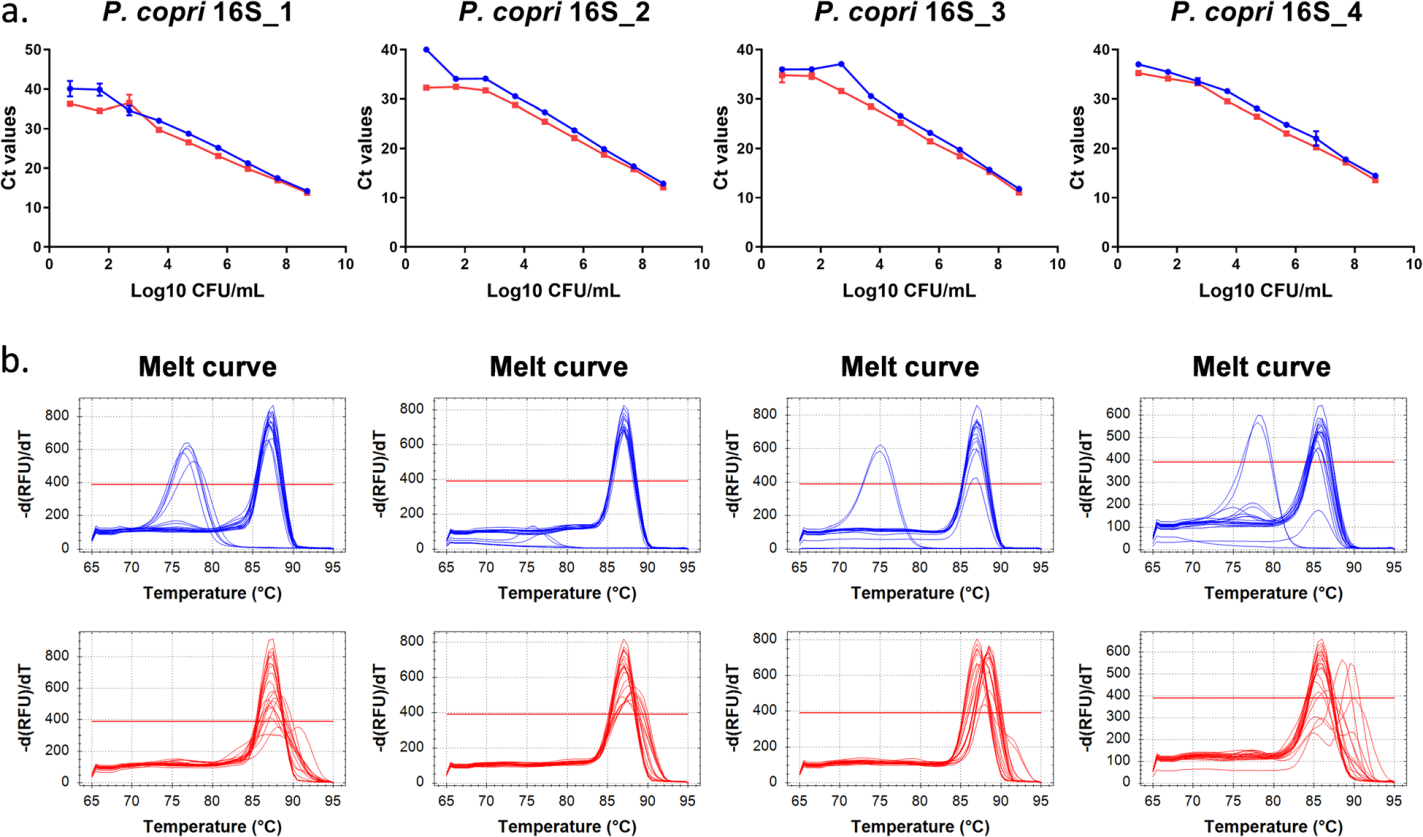
Performance of newly developed *P. copri* 16S primers under standard PCR conditions. **a** Standard curves and **b** melt curves (blue: *P.copri* only and red: murine faeces spiked with *P. copri*)

**Fig. 3:**
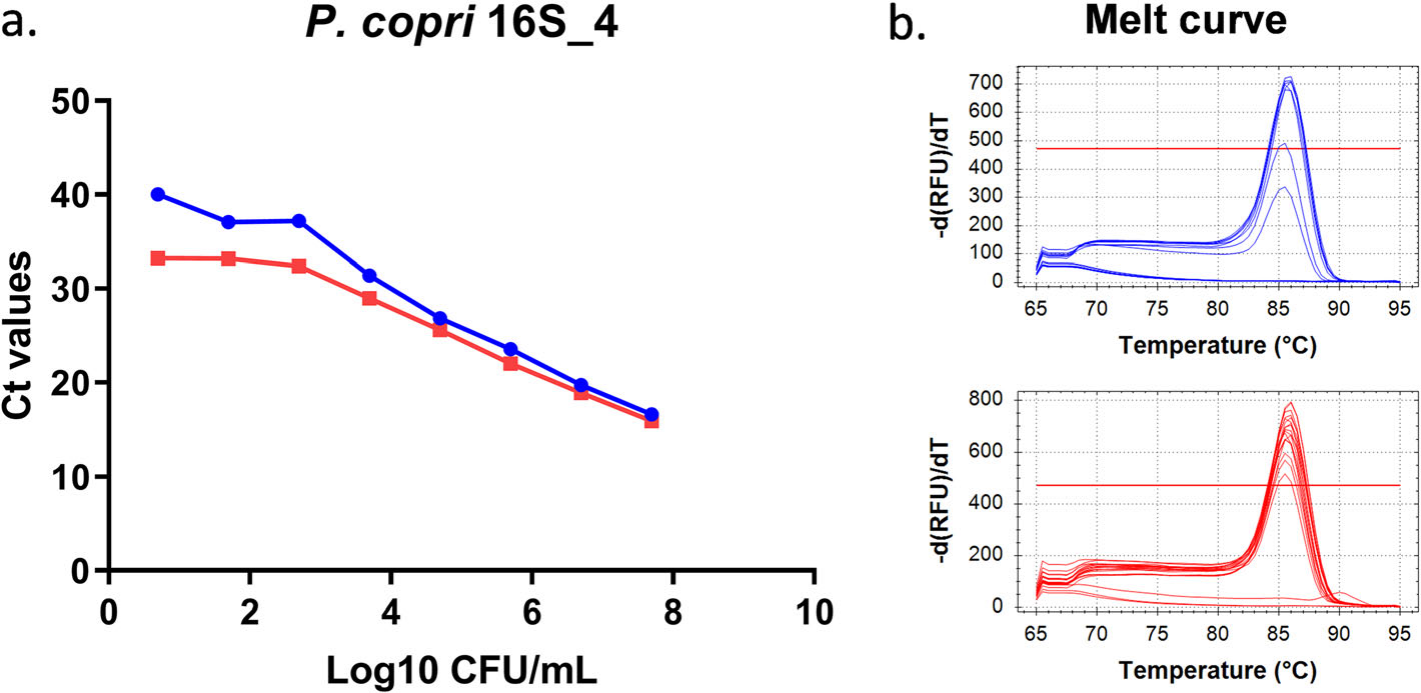
Performance of newly developed *P. copri* 16S primers under stringent PCR conditions. **a** Standard curves and **b** melt curves (blue: *P. copri* only and red: murine faeces gDNA samples spiked with different concentrations of *P. copri*)

To increase specificity and sensitivity, a number of primer pairs targeting the *P. copri* 16S rRNA non-conserved regions were designed (supplementary Figure 2). As for the original *P. copri* 16S rRNA primers described above, there were a number of melt curve peaks in the mixed murine faeces at lower *P. copri* concentrations indicating binding of the primers to aspecific sequences (Fig. 2). As long amplicon lengths compromise the amplification efficiency when short cycling times are used, only the P.copri_16S_4 primer pair was tested further under more stringent PCR conditions (15 s annealing/extension time instead of 60 s and increasing annealing/extension temperature from 60 to 62 degrees). Substantial improvement in sensitivity and specificity was observed for P.copri_16S_4 when more stringent PCR conditions were applied. For P.copri_16S_4 primers, the specificity was increased in the lower bacterial concentrations down to 10^3^ CFU/mL with almost no amplification of aspecific products (all peaks at the same temperature in melt curves) present in the faeces (Fig. 3).

### Assessment of *P. copri* genome-specific primers

**Fig. 4:**
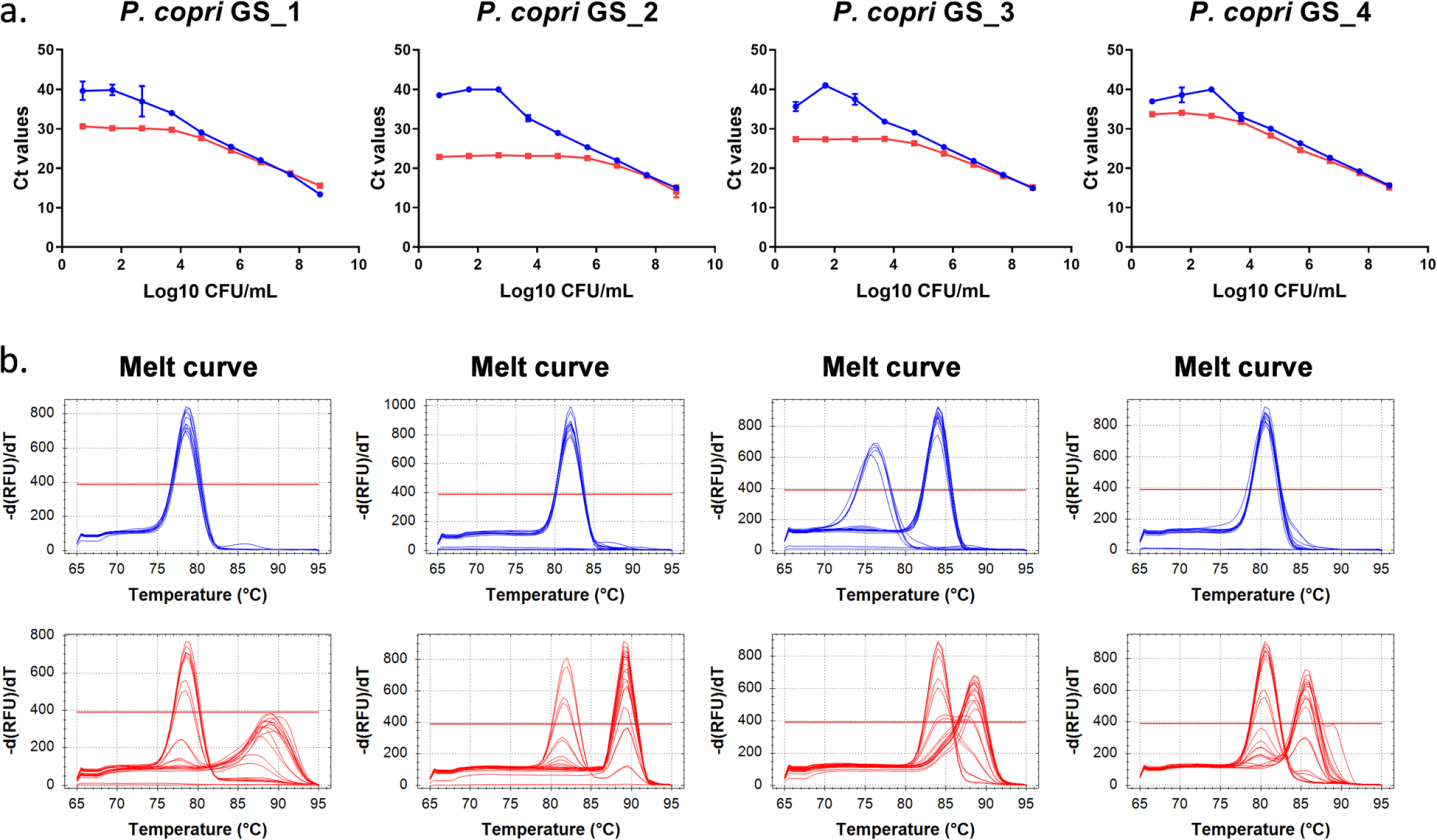
Performance of newly developed *P. copri* genome specific primers under standard PCR conditions. **a** Standard curves and **b** melt curves (blue: *P.copri* only and red: faeces spiked with *P. copri*)

To avoid aspecificity due to binding of the very conserved *P. copri* 16S rRNA gene primers to 16S rRNA genes of closely related bacterial species (e.g. other *Prevotella* species), primers targeting genes unique for the *P. copri* genome were also designed using the published genome sequence of the DSM18205 reference strain (for a list of *P. copri*-specific genes see [17]). Surprisingly however, aspecific product amplification (indicated by additional melt curve peaks at higher temperatures) was still observed in the faeces samples spiked with no or low amounts of bacteria. Figure 4 shows the standard curves generated from serial dilutions of CFU 10^1^–10^9^/mL for four *P. copri* specific gene primer sets. Interestingly, *P. copri* genome specific (GS) primers did thus not decrease the background under standard PCR conditions.

**Fig. 5:**
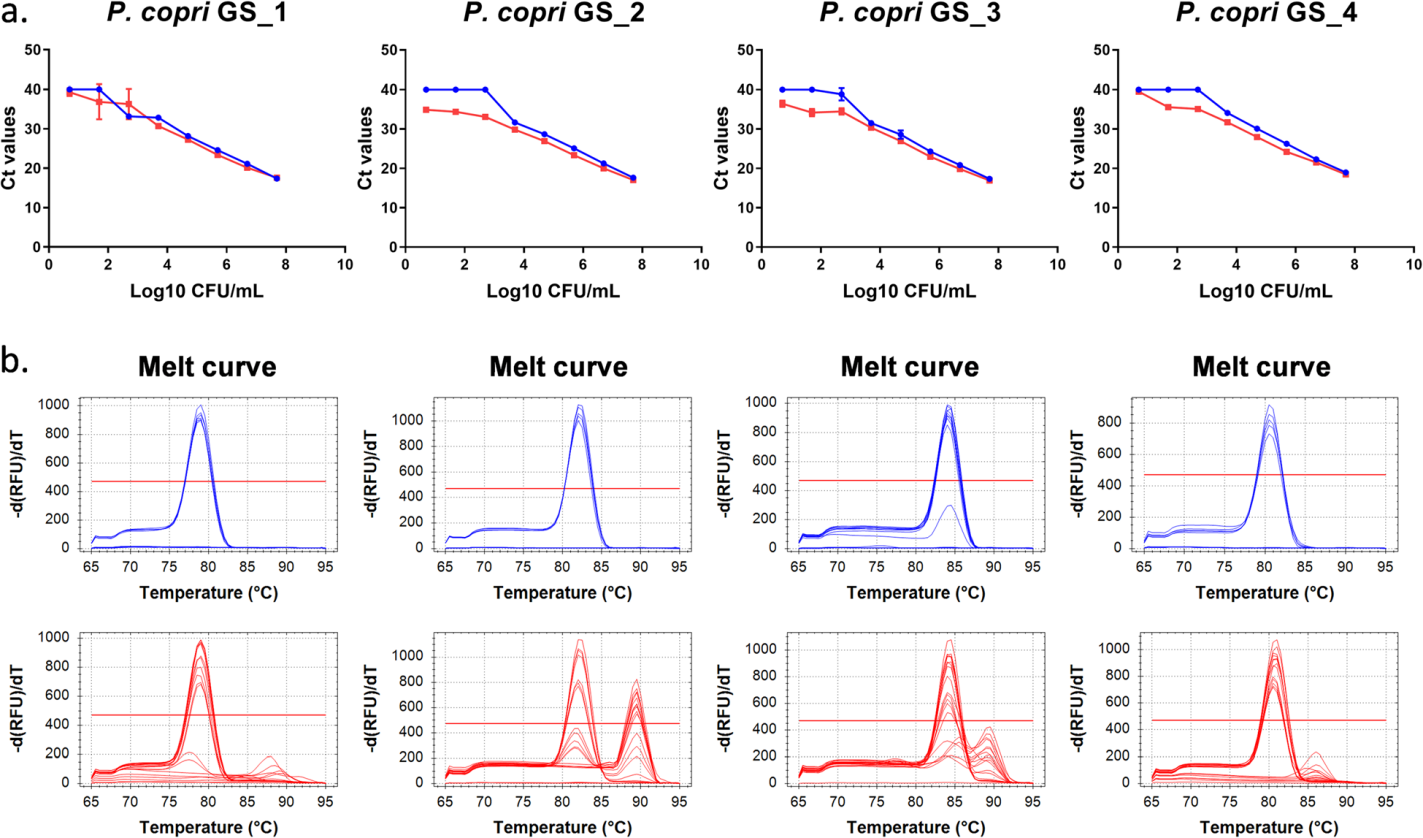
Performance of newly developed *P. copri* genome specific primers under stringent PCR conditions. **a** Standard curves and **b** melt curves (blue: *P.copri* only and red: faeces spiked with *P. copri*). Primer pairs P.copri_GS_1 and P.copri_GS_4 performed better with the new PCR conditions

Substantial improvement in sensitivity and specificity of the genome specific primers was observed when more stringent PCR conditions were used (15 s annealing/extension time instead of 60 s and increasing annealing/extension temperature from 60 to 62 degrees). For primer pair P.copri_GS_1 and P.copri_GS_4, the specificity was increased in the lower bacterial concentrations down to 10^3^ CFU/mL with almost no amplification of aspecific products (all peaks at same temperature in melt curves) present in the faeces (Fig. 5).

### The use of different qPCR mastermixes affects sensitivity and specificity

**Fig. 6:**
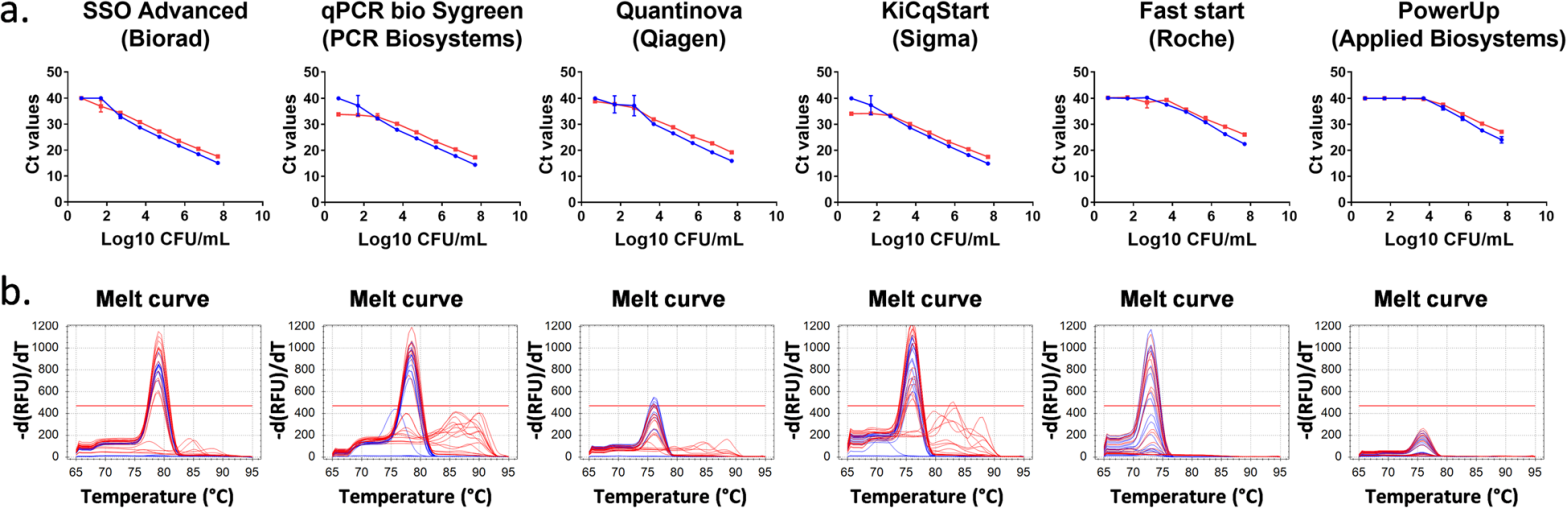
Performance of different commercially available SYBR Green mixes. Standard curves (**a**) and melt curves (**b**) for both pure *P. copri* samples (blue) and faeces spiked with different amounts of *P. copri* bacteria (red)

A number of commercially available qPCR SYBR green mixes was tested for sensitivity and specificity using the P.copri_GS_1 primers (Fig. 6). The specificity, as determined by the number of peaks in the melting curves, was highest for PowerUp SYBR Green Master Mix, FastStart SYBR Green Master Mix, Quantinova SYBR Green PCR kit and SsoAdvanced Universal SYBR Green Supermix (single peak in the melt curves), while amplification of aspecific products could be observed for the qPCRBIO SyGreen Mix and KiCqstart SYBR Green qPCR Ready Mix (multiple peaks in melt curves towards the lower concentrations of bacteria in the presence of faeces). On the other hand, the sensitivity, as determined by the lowest CFU/mL concentration before the standard curves reached a plateau, was highest for SsoAdvanced Universal SYBR Green Supermix, Quantinova SYBR Green PCR Kit, qPCRBIO SyGreen Mix and KiCqstart SYBR Green qPCR Ready Mix (10^2^ CFU/mL), while FastStart SYBR Green Master Mix and PowerUp SYBR Green Master Mix showed the lowest sensitivity (10^4^ CFU/mL). Taken together, SsoAdvanced Universal SYBR Green Supermix, the mix that had been used in the experiments above, resulted in the highest specificity and sensitivity.

### Correlation between the relative abundance of *P. copri* determined by qPCR and 16S rRNA MiSeq sequencing

**Fig. 7:**
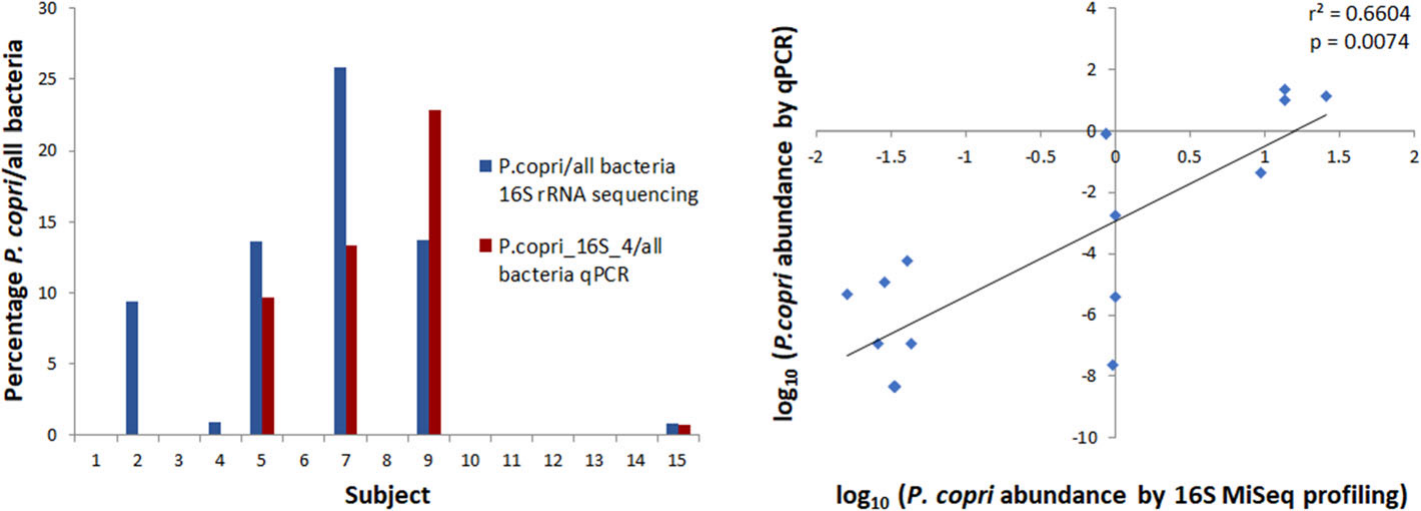
*P. copri* abundance in human faeces determined by 16S rRNA gene sequencing and by 16S rRNA qPCR using the P. copri_16S_4 primer set and universal 16S primers (left: bar graph; right: scatter plot). There is a correlation (*r*^2^ = 0.66, *p* < 0.01, *n* = 15) between the relative abundance of *P. copri* determined by qPCR and the relative abundance determined by 16S rRNA gene sequencing

To determine if our qPCR-based quantification method is comparable to 16S rRNA profiling by Illumina MiSeq sequencing, we measured relative abundance of *P. copri* in 15 human faeces samples using both methods (Fig. 7). A clear correlation was found between the MiSeq-based method and qPCR using the 16S_4 primers (*r*^2^ = 0.6604, *p* = 0.0074). In contrast, qPCR using the P.copri_GS_1 and P.copri_GS_4 primers on the human faeces samples yielded no detectable signal (data not shown).

### In silico validation of primer binding

To determine why no signal was detected in the human stool samples using the P.copri_GS_1 and P.copri_GS_4 primers, we analysed in silico binding of the different primer pairs to the sequenced genomes of 114 *P. copri* strains obtained from the PATRIC database. In agreement, the P.copri_GS_1 and P.copri_GS_4 primers only bound to the reference strain DSM18205, while the P.copri_16S_4 primers bound to 83 of the 114 strains (Supplementary Table 1). We also tested the Scher primers [12], which could only bind to 35 *P. copri* strains, including the reference strain DSM18205. Another study recently published a Taqman-probe based qPCR method to detect the presence of *P. copri* in a cohort of Australian women [16]. We tested in silico binding of their 16S rRNA-based primers in the genomes of the *P. copri* strains, and found they could detect the large majority (107/114) of strains.

To test specificity of our P.copri_16S_4 primers and those developed by Scher et al. and Gray et al. in related species of the *Prevotella* genus, we analysed in silico binding to the genomes of a range of representative *Prevotella* species (obtained from the PATRIC database), as represented in the phylogenetic tree (Supplementary Figure 1). While our P.copri_16S_4 primers and the primers developed by Scher et al. yielded no predicted PCR products in any of the tested genomes, the Gray primers yielded nearly identical products of 55 bp in five of the seven non-copri *Prevotella* species. For the Gray reverse primer, 19 out of 20 bases were direct matches (Supplementary Figure 2), while e.g. 17 out of 19 bases (including the 14 bases at the 3′ end) of the Gray forward primers were perfectly matched to the genomes of *P. jejuni, veroralis, histicola* and *melanogenica*. However, the Gray et al. 14 bp Taqman probe contains two mismatches between *P. copri* and the *Prevotella* species mentioned above, so the use of the Gray primers in combination with the TaqMan probe would improve species specificity. Overall, our P.copri_16S_4 primers provide a good balance of detecting a wide range of *P.copri* strains in a quantitative manner, without off-target detection of other *Prevotella* genus species while the P.copri_GS_1 and GS_4 primers are highly specific for only the *P.copri* reference DSM 18205 strain.

### No in vitro cross-reactivity with closely related *Prevotella* species for the newly designed *P. copri* primer sets

**Fig. 8:**
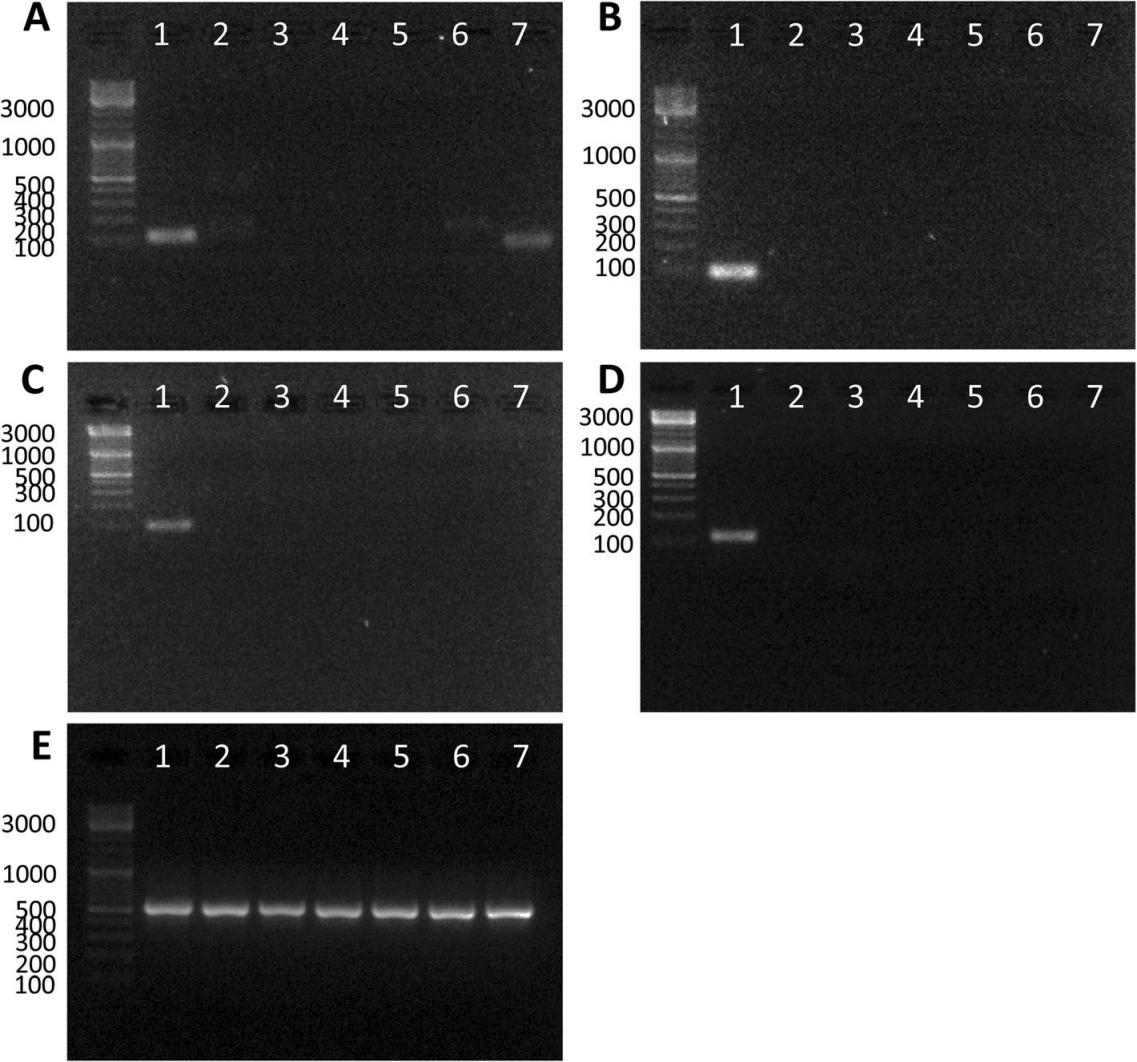
Specificity of the primer sets validated with related *Prevotella* species. **a** primer set P. copri Scher et al. [12]; **b** primer set P. copri_16S_4: **c** primer set P. copri_GS_1; **d** primer set P. copri_GS_4 and **e** primer set *Prevotella* genus (Matsuki et al. 2002, [12]) as positive control. Lanes indicate: 1: *P. copri*; 2: *P. salivae*; 3: *P. paludivivens*: 4: *P. jejuni*; 5: *P. melaninogenica*; 6: *P. histicola*; 7: *P. veroralis*

As recommended by Balakrishnan et al. [18], the specificity of the primer sets from Scher et al. [12] (Fig. 8a), P.copri_16S_4 (Fig. 8b), P.copri_GS_1 (Fig. 8c) and P.copri_GS_4 (Fig. 8d) was tested by qPCR on DNA of representative species phylogenetically most closely related to *P. copri* (Supplementary Figures 1 and 3). While the primers from Scher showed cross-reactivity with other *Prevotella* species such as *P. veroralis*, the newly designed primers specifically and exclusively amplified *P. copri* DNA.

## Discussion

In contrast to traditional culture-dependent methods used in bacterial identification, real-time PCR is fast, cost-effective, quantitative and highly sensitive and is thus increasingly used in clinical diagnostics [19]. Real-time PCR assays, however, are limited by the quality of the primers which must be sensitive enough to target the organism of interest, yet specific enough to exclude all others. The primers for the gut bacterium *P. copri* currently used in the literature [12] work well on pure *P. copri* isolates. Their sensitivity and specificity is however lower in complex samples such as faeces, that contain DNA from e.g. plant, animal and microbial cells present in food, host cells and both living and degraded (micro) organisms, some of which - such as other *Prevotella* species- are closely related to the species to be identified. In this work, three primer pairs (P.copri_16S_4, P.copri_GS_1 and P.copri_GS_4) were identified that have up to 10x higher specificity and sensitivity in complex biological samples than the primer-only PCR currently cited in the literature [12]. These results highlight the need to test standards in complex samples to ensure accuracy in quantification and eliminate deviations due to other DNA present. Cross-reactivity with the closest related *Prevotella* species was absent for these newly designed primers while this was not the case for the primer-only PCR currently cited in the literature [12] and recently developed primers by Gray *et al* [16]. *P. copri* abundance in human faeces determined by qPCR using the newly designed P.copri_16S_4 primers was shown to correlate well with that obtained by 16S rRNA sequence bacterial profiling, while the P.copri_GS_1 and GS_4 did not yield any products in the human faeces samples. In silico analysis showed that the P.copri_16S_4 primers bound to 83 of 114 *P. copri* strains (compared to only 35 for the Scher primers) while the primer sets targeting genes unique to (P.copri_GS_1 and GS_4) only bound to the reference strain DSM18205. These data for the non 16S gene specific primers are in line with the finding that *P. copri* is one of the most plastic gut colonisers with high subspecies genetic diversity between different subjects [20]. The Gray et al. 16S rRNA primers can potentially detect a wider range of *P. copri* strains, but at the cost of likely detecting non-*copri Prevotella* species. Therefore, the use of the specific TaqMan probe is most likely required to obtain species specificity. Our 16S rRNA primers have the advantage that they show species specificity without the use of costly TaqMan probes and are compatible with standard SYBR green-based detection methods. The new P.copri_16S_4 primers are thus useful for picking up a wide range of *P. copri* susbspecies (eg. in environmental studies) while the P.copri GS_1 and GS4 primers can be used to specifically detect the DSM18205 reference strain, allowing differential detection and relative quantification of indigenous and administered (eg. to human subjects or mice) *P. copri* strains. The methodology described here may be useful in the detection of other bacterial species in complex samples. In addition, it was shown that SYBR green PCR mixes have an influence on sensitivity and specificity, with Biorad SsoAdvanced performing the best under our test conditions.

## Conclusions

This qPCR-based method will substantially improve the sensitivity and specificity of *P.copri* identification and quantification compared to the primer-only qPCR method most commonly cited in the literature [12]. This method also enables the accurate detection of a wider range of *P. copri* subspecies and of *P. copri* DSM18205 specifically. Moreover, the methodology described here can be used as a model to detect and quantify other bacterial species in complex samples.

## Methods

### Cultivation of *P. copri*

*P. copri* strain CB7 (DSM 18205, DSMZ GmbH) was cultured on Schaedler agar (Sigma-Aldrich) plates at 37 °C in an anaerobic N_2_/CO_2_/H_2_ atmosphere (85/10/5%) maintained by a Whitley DG250 workstation. For counting of colony forming units (CFU) serial dilutions of the harvested bacteria were plated on Schaedler agar plates.

### Collection of murine faeces

Faeces were collected from 12 week old healthy male wild type C57BL/6 J mice commercially obtained from Janvier Labs (Marseille, France) and housed in a specific pathogen free animal facility.

### gDNA extraction

*P. copri* DSM18205 bacteria were harvested from agar plates with sterile loops, washed by suspending in 0.9% NaCl and spinning down, resuspended in 0,9% NaCl and a fraction was plated out for colony counting. For the pure *P. copri* sample, a volume corresponding to 10^9^ bacteria was centrifuged at 3000 rpm and resuspended in lysis buffer. For the *P. copri*-spiked murine faeces samples, faeces were collected from C57BL/6 J mice (Janvier), homogenized using a Qiagen Tissuelyser II and aliquoted. Each aliquot was spiked with 10 uL of up to eight 10-fold dilutions of *P. copri* bacteria in 0.9% NaCl with 10^1−^ 10^9^ CFU/mL. gDNA from bacteria only or faeces spiked with different amounts of bacteria was then prepared according to the manufacturor’s instructions using the QIAamp PowerFecal DNA kit (Qiagen, Hilden, Germany). Samples were added to lysis buffer and incubated at 65 °C for 10 min and disrupted and homogenized in a Bead Tube containing garnet beads, using a TissueLyser II (Qiagen) prior to DNA extraction. DNA concentrations and purity were measured using a Nanodrop spectrophotometer (Nanodrop Technologies, Wilmington, DE, USA).

### Primer design

16S ribosomal RNA (16S rRNA) gene sequences for the *P. copri* type strain were obtained from NCBI (GenBank accession AB064923). This sequence was aligned with the 7 closest phylogenetically related *Prevotella* species (as determined by blasting the *P. copri* reference sequence). For the phylogenetic tree, 16S rRNA sequences were aligned and a phylogenetic tree was calculated with 1000 bootstraps using ClustalX. The phylogenetic tree was visualised using NJplot (Supplementary Figures 1 and 2). Primers were designed using primer 3 software targeting either the *P. copri* 16S rRNA gene or *P. copri* specific genes [17]: glycosyl transferase, group 1 (PREVCOP_06806) for primer set P.copri_GS_1, RNA polymerase ECF-type sigma factor (PREVCOP_06715) for primer set P.copri_GS_2, CAAX amino protease family protein (PREVCOP_06538) for primer set P.copri_GS_3 and metalloprotease domain protein, M6 family (PREVCOP_04242) for primer set P.copri_GS_4 (for primer details see Table 1 [12, 21]). A BLAST [22] search ensured that the primers were not hitting other targets.

**Table 1:** Primers used in qPCR studies

Primer name	Sequence	#nucleotides	Amplicon length (bp)	Target gene	Reference
Universal 16S, F	ACTCCTACGGGAGGCAGCAGT	21	192	16S rRNA	Scher et al., 2013 [12]
Universal 16S, F	ATTACCGCGGCTGCTGGC	18			
*P. copri* genus 16S, F	CACRGTAAACGATGGATGCC	20	516	16S rRNA	Matsuki et al., 2002
*P. copri* genus 16S, R	GGTCGGGTTGCAGACC	16			
*P. copri* genome specific, F	CCGGACTCCTGCCCCTGCAA	20	106	FimB/Mfa2 family fimbrial subunit	Scher et al., 2013 [12]
*P. copri* genome specific, R	GTTGCGCCAGGCACTGCGAT	20		PREVCOP_RS00015	
*P. copri* 16S, 1F	ACATCGAAAGCTTGCTTTTG	20	409	16S rRNA	This publication
*P. copri* 16S, 1R	CAAAAAGCCTCACGAGGCTC	20		AB064923	
*P. copri* 16S, 2F	ACCACTTGGGGATAACCTTG	20	347	16S rRNA	This publication
*P. copri* 16S, 2R	TACATGCAAAAAGCCTCACGAGGC	24		AB064923	
*P. copri* 16S, 3F	TCTCTAGAAGACATCTGAAAGA	22	446	16S rRNA	This publication
*P. copri* 16S, 3R	CAGTGCAGACGTTGAGCGT	19		AB064923	
*P. copri*16S, 4F	CGAAAGCTTGCTTTTGATGG	20	86	16S rRNA	This publication
*P. copri* 16S, 4R	CGCAAGGTTATCCCCAAGT	19		AB064923	
*P. copri* genome specific, 1F	TTTTGCTGTAGGAGGGGTTG	20	96	glycosyl transferase, group 1	This publication
*P. copri* genome specific, 1R	GGGCTGCATAAAGCAAAGAC	20		PREVCOP_06806	
*P. copri* genome specific, 2F	AGCCGAGATATCGTGAGTGG	20	137	RNA polymerase ECF-type sigma factor	This publication
*P. copri* genome specific, 2R	TGAACAGCTGTATGCCGAAG	20		PREVCOP_06715	
*P. copri* genome specific, 3F	AGTTTGTCAATGCCCTCCTG	20	141	CAAX amino protease family protein	This publication
*P. copri* genome specific, 3R	CATCGCTCTGAGGCATGATA	20		PREVCOP_06538	
*P. copri* genome specific, 4F	TCGCTGACATGAGCGATAAC	20	109	metalloprotease domain protein, M6 family	This publication
*P. copri* genome specific, 4R	CCGTTGGCACTACCTTCATT	20		PREVCOP_04242	

### Real-time quantitative PCR (qPCR)

10-fold serial dilutions in DEPC water were made from 156 ng/μL pure *P. copri* gDNA (equivalent to 10^9^ to 10^1^ CFU/mL). For gDNA of murine faeces spiked with similar amounts of *P. copri* as for the pure *P. copri* DNA, 20 ng gDNA was used in each qPCR amplification. Samples were run in duplicate on the same plate for the same gene. Detection of the PCR product was carried out by the CFX384 Touch™ Real-Time PCR system (Biorad, Hercules, CA, USA) using the DNA-binding dye SYBR Green I (SsoAdvanced Universal SYBR Green Supermix unless stated otherwise). The standard 2-step qPCR cycling conditions were as follows: pre-denaturation of 98 °C for 3 min, 40 cycles of denaturation of 95 °C for 15 s and an annealing/extension step at 60 °C for 1 min. Stringent PCR conditions refer to 15 s annealing/extension time (instead of 60 s) and increasing annealing/extension temperature from 60 to 62 degrees. ‘No template’ controls (containing DEPC water) were included in each run. The following SYBR Green mastermixes were tested: SsoAdvanced Universal SYBR Green Supermix (Cat No. 172–5271, Bio-Rad), qPCRBIO SyGreen Mix (Cat No. PB20.14, PCR Biosystems), PowerUp SYBR Green Master Mix (Cat No. A25741, Applied Biosystems), FastStart SYBR Green Master (Cat. No. 04 673 484 001, Roche), KiCqStart SYBR Green qPCR Ready Mix (Cat. No. KCQS00, Sigma-Aldrich) and QuantiNova SYBR Green PCR Kit (Cat. No. 208059, Qiagen). For cross-reactivity testing, 20 ng DNA of *P. copri* strain CB7 (strain DSM18205), *P. salivae* (DSM15606), *P. paludivivens* (DSM17968), *P. jejuni* (DSM26989), *P. melaninogenica* (DSM7089), *P. histicola* (DSM19854) and *P. veroralis* (DSM19559) (all supplied by DSMZ GmbH) was amplified under stringent PCR conditions as above and was run on a 2% agarose gel (Sigma-Aldrich) adjacent to the DNA ladder GeneRuler DNA Ladder Mix (Thermofisher scientific).

### 16S RNA sequencing

Faecal samples were collected in duplicate from 15 subjects (8 male, 7 female) aged 26,9 ± 4,3 years with normal body mass indices (BMI) of 23,9 ± 3,9 kg/m^2^, and immediately stored at -80 °C until analysed. Faecal DNA was extracted using the QIAamp Powerfaecal DNA kit (Qiagen), including a bead beating step. Faecal amplicon DNA concentrations were quantified using a Quant-iT dsDNA Assay Kit, High Sensitivity (Life Technologies) using the Fluoroskan™ Microplate Fluorometer (Life Technologies, Carlsbad, CA, USA). The V4 region of 16S rRNA genes was amplified by PCR with forward primer 5′ TCGTCGGCAGCGTCAGATGTGTATAAGAGACAGGTGCCAGCMGCCGCGGTAA and reverse primer 5′ GTCTCGTGGGCTCGGAGATGTGTATAAGAGACAGGGACTACHVG

GGTWTCTAAT [23], and extended with Illumina (Illumina Inc., San Diego, CA, USA) adapter sequences and unique dual indexes to tag each PCR product, according to the 16S-protocol provided by Illumina. In short, PCRs contained 0.2 μM primers, 12.5 ng template DNA and 12.5 μL of 2 × KAPA HiFi HotStart Ready Mix kit (KAPA Biosystems, Woburn, MA, USA) in a reaction volume of 25 μL. Thermal cycling conditions were as follows: initial denaturation at 95 °C for 3 min, 25 cycles of denaturation at 95 °C for 30 s, annealing at 55 °C for 30 s, and extension at 72 °C for 30 s and a final step of 72 °C for 5 min. Purification of the products was carried out with the Agencourt AMPureXP Kit (Beckman Coulter, Miami, FL, USA). Adapters and unique dual indexes were then attached to each sample using a Nextera XT index kit (Illumina), after which a second clean-up step was performed using the AMPureXP Kit (Beckman Coulter). The size of the PCR amplicons was verified using 0.1% agarose gel electrophoresis. No visible bands were observed for the negative extraction controls. 4 pM of the amplicons and 5% of PhiX control v3 (internal control) were collected into a single tube. Paired-end sequencing with a read length of 2 × 300 bp was performed on a Miseq Instrument (Illumina) using a Miseq v2 reagent kit (Illumina). The sequence analysis, finally, was carried out using the free software package Quantitative Insights into Microbial Ecology (QIIME).

## Supplementary Information

**Additional file 1: Figure S1.** Phylogenetic tree of the species closest related to *Prevotella copri*.

**Additional file 2: Figure S2.** CLUSTAL O (1.2.4) multiple sequence alignment. Primers are indicated by arrows.

**Additional file 3: Figure S3.** Specificity of the primer sets validated with related *Prevotella* species (cf. Fig. 8) with inverted alternative exposures. A: primer set P.copri Scher et al. [12]; B: primer set P. copri_16S_4: C: primer set P.copri_GS_1; D: primer set P.copri_GS_4 and E: primer set *Prevotella* genus (Matsuki et al. 2002, [12]) as positive control. Lanes indicate: 1: *P. copri*; 2: *P. salivae*; 3: *P. paludivivens*: 4: *P. jejuni*; 5: *P. melaninogenica*; 6: *P. histicola*; 7: *P. veroralis*.

**Additional file 4: Table S1.** In silico analysis of primer binding to 114 *P. copri* strains obtained from the PATRIC database. Columns contain the following information: genome name, PATRIC genome ID, whether both forward and reverse primers bind to strain (for P.copri_GS_1, P.copri_GS_4, P.copri_16S_4, *P. copri* primers used in Scher et al. [12] and Gray et al. [16]); size of genome of *P. copri* strain and number of contigs.

## Data Availability

The datasets used and analysed in the current study are available from the corresponding author on reasonable request.

## Abbreviations

*P. copri*: *Prevotella copri*
CFU: Colony forming units
qPCR: Quantitative Polymerase Chain Reaction
16S rRNA: 16S ribosomal RNA
gDNA: Genomic DNA
