# Supplementary figures and images for: Development of a real-time quantitative PCR method for detection and quantification of Prevotella copri
Source: BMC Microbiol. 2021 Jan 11;21:23. doi: 10.1186/s12866-020-02063-4 (PMC7798335; doi:10.1186/s12866-020-02063-4)

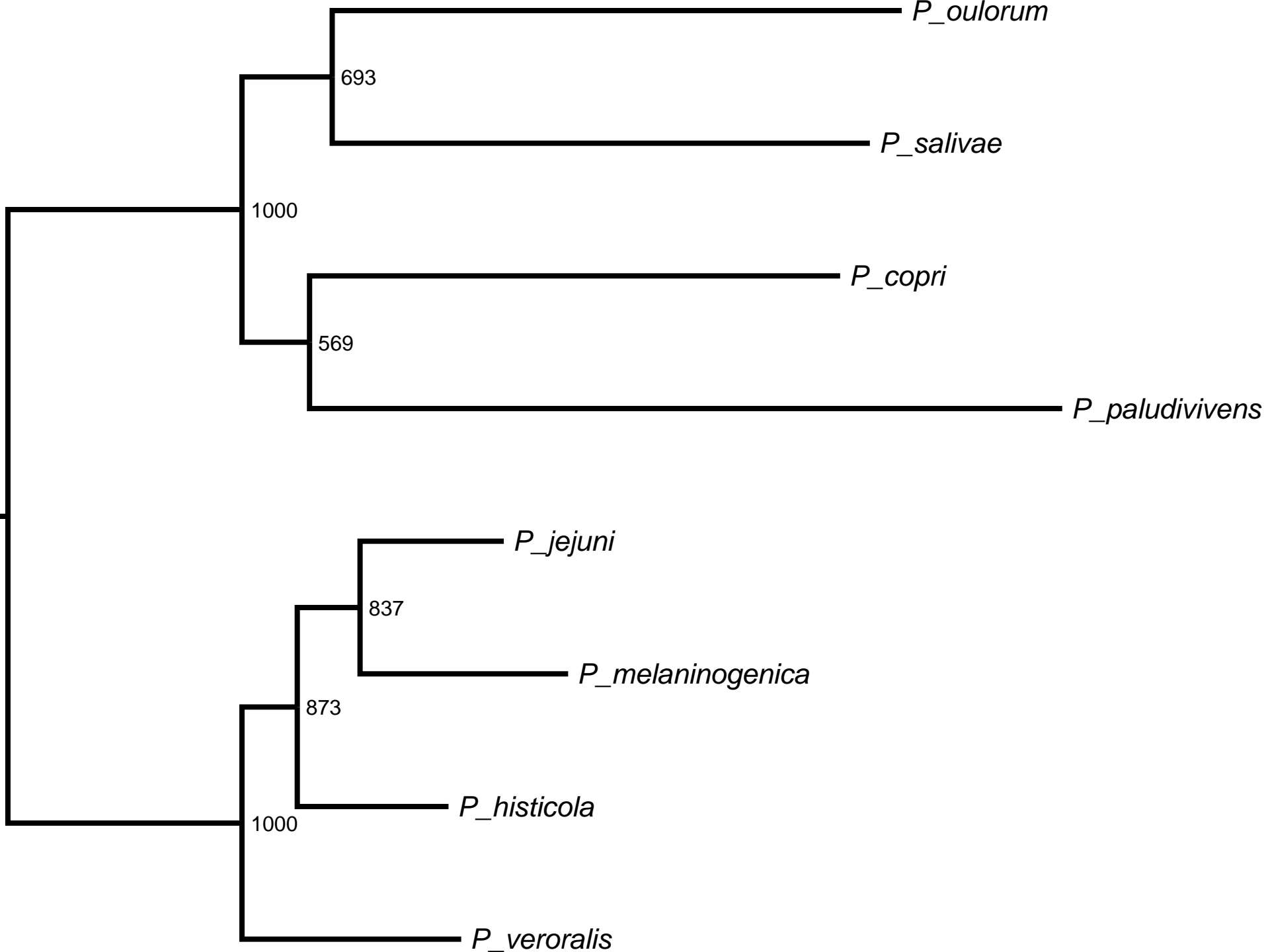

0.005

Supplement: Supplementary file 1 — Additional file 1: Figure S1. Phylogenetic tree of the species closest related to Prevotella copri. [file 12866_2020_2063_MOESM1_ESM.pdf]

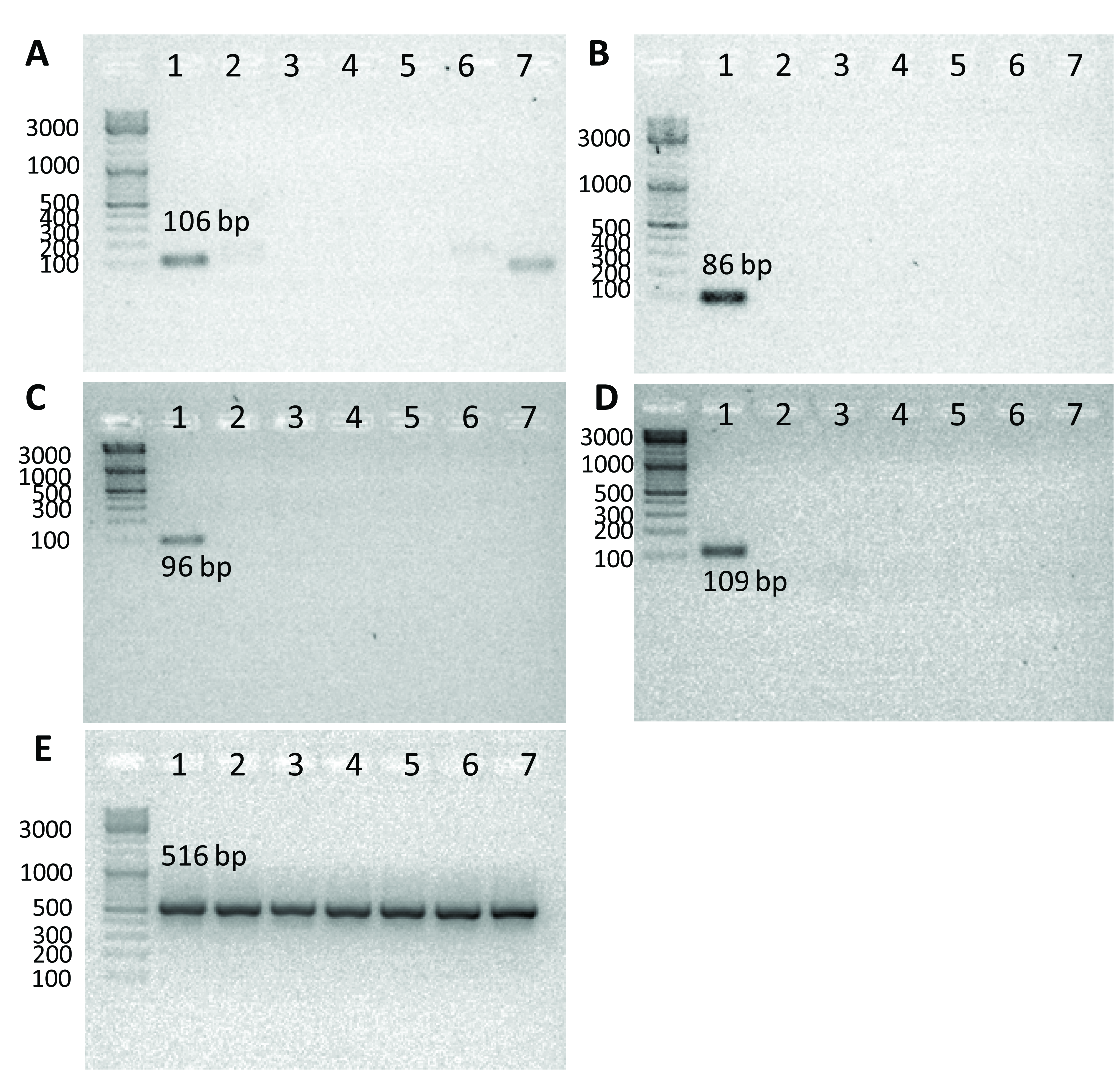

Supplement: Supplementary file 3 — Additional file 3: Figure S3. Specificity of the primer sets validated with related Prevotella species (cf. Fig. 8) with inverted alternative exposures. A: primer set P.copri Scher et al. [12]; B: primer set P. copri_16S_4: C: primer set P.copri_GS_1; D: primer set P.copri_GS_4 and E: primer set Prevotella genus (Matsuki et al. 2002, [12]) as positive control. Lanes indicate: 1: P. copri; 2: P. salivae; 3: P. paludivivens: 4: P. jejuni; 5: P. melaninogenica; 6: P. histicola; 7: P. veroralis. [file 12866_2020_2063_MOESM3_ESM.tif]

## Slide 1
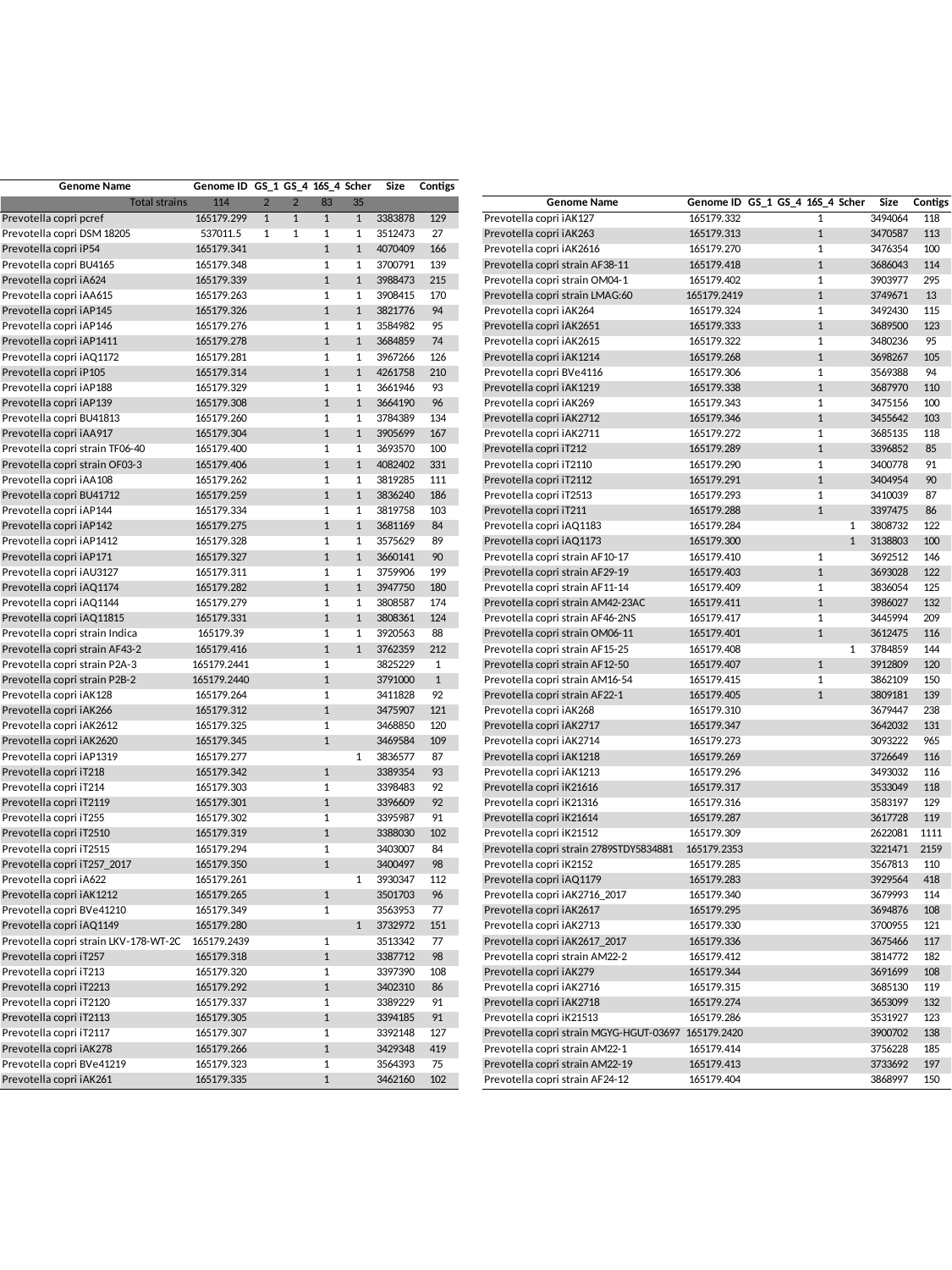

Supplement: Supplementary file 4 — Additional file 4: Table S1. In silico analysis of primer binding to 114 P. copri strains obtained from the PATRIC database. Columns contain the following information: genome name, PATRIC genome ID, whether both forward and reverse primers bind to strain (for P.copri_GS_1, P.copri_GS_4, P.copri_16S_4, P. copri primers used in Scher et al. [12] and Gray et al. [16]); size of genome of P. copri strain and number of contigs. [file 12866_2020_2063_MOESM4_ESM.pptx]
