# Supplementary material for: Development of a real-time quantitative PCR method for detection and quantification of Prevotella copri
Source: BMC Microbiol. 2021 Jan 11;21:23. doi: 10.1186/s12866-020-02063-4 (PMC7798335; doi:10.1186/s12866-020-02063-4)
Supplement: Supplementary file 2 — Additional file 2: Figure S2. CLUSTAL O (1.2.4) multiple sequence alignment. Primers are indicated by arrows. [file 12866_2020_2063_MOESM2_ESM.pdf]

```
                                                4F
                                 1F
P_copri            ----agagtttgatcctggctcaggatgaacgctagctacaggcttaacacatgcaagtc  56
P_jejuni           -----------------------ggatgaacgctagctacaggcttaacacatgcaagtc  37
P_veroralis        ----agagtttgatcctggctcaggatgaacgctagctacaggcttaacacatgcaagtc  56
P_histicola        ----------------tggctcaggatgaacgctagctacaggcttaacacatgcaagtc  44
P_melaninogenica   ----agagtttgatcctggctcaggatgaacgctagctacaggcttaacacatgcaagtc  56
P_oulorum          atggagagtttgatcctggctcaggatgaacgctagctacaggcttaacacatgcaagtc  60
P_salivae          ----agagtttgatcctggctcaggatgaacgctagctacaggcttaacacatgcaagtc  56
P_paludivivens     ----agagtttgatcctggctcaggatgaacgctagctacaggcttaacacatgcaagtc  56
                                          *************************************

P_copri            gaggggaaacgacatcgaaagcttgcttttgatgggcgtcgaccggcgcacgggtgagta  116
P_jejuni           gaggggaaacggcatttagtgcttgcactgaatggacgtcgaccggcgcacgggtgagta  97
P_veroralis        gaggggaaacggcattgagtgcttgcactgaatggacgtcgaccggcgcacgggtgagta  116
P_histicola        gaggggaaacggcattaagtgcttgcacttttggacgtcgaccggcgcacgggtgagta  104
P_melaninogenica   gaggggaaacggcattgagtgcttgcactctttggacgtcgaccggcgcacgggtgagta  116
P_oulorum          gaggggaaacgatgacgagagcttgctct-cttaggcgtcgaccggcgcacgggtgagta  119
P_salivae          gaggggaaacgacattg-aagcttgcttc-gatgggcgtcgaccggcgcacgggtgagta  114
P_paludivivens     gaggggtaacgaga-gggaagcttgcttt-ccttgtcgacgaccggcgcacgggtgagta  114
                   ****** ****        ******      * * ** ********************
                                                    4R
                                                 2F
P_copri            acgcgtatccaacctgcccaccacttggggataaccttgcgaaagtaagactaataccca  176
P_jejuni           acgcgtatccaacctacccattactgtgggataacctgccgaaaggcagactaataccgc  157
P_veroralis        acgcgtatccaaccttcccataactaagggataacctgccgaaaggcagactaatacctt  176
P_histicola        acgcgtatccaaccttcccatgactaagggataacctgccgaaaggcagactaatacctt  164
P_melaninogenica   acgcgtatccaaccttcccattactgtgggataacctgccgaaaggcagactaataccgc  176
P_oulorum          acgcgtatccaacctgcccattacaggggaataaccttgcgaaagtaagactaatgcccc  179
P_salivae          acgcgtatccaacctgcctctgactaagggataacccggcgaaagtcggactaatacctt  174
P_paludivivens     acgcgtatccaacctgcccacaactagagaataaccttgcgaaagtaagactaatgctct  174
                   ************** **     **     * ******    ******    ******* *
                                          3F
P_copri            atgatatctctagaagacatctgaaagagattaaagatttatcggtgatggatggggatg  236
P_jejuni           atagtcttcgatgacggcatcagatttgaagtaaagatttatcggtaatggatggggatg  217
P_veroralis        atgtaattcttagatgacatcagaagagaatgaaagatttatcggttatggatggggatg  236
P_histicola        atggtcttcactgacggcatcagatgtgaagtaaagatttatcggttatggatggggatg  224
P_melaninogenica   atagtcttcgatgacggcatcagatttgaagtaaagatttatcggtaatggatggggatg  236
P_oulorum          atggtattctcagcagacatctaatgaggattaaagatttatcggtaatggatggggatg  239
P_salivae          atgaggttttcagcagacatctaacgaaaacgaaagatttatcggtcagtgatggggatg  234
P_paludivivens     ataatttccttagatggcatctgacgaggaataaagatttatcggttatggatggggatg  234
                   **     *      *  * ****  *      *  ************** *  **********

P_copri            cgtctgattagcttgttggcggggtaacggcccaccaaggcgacgatcagtaggggttct  296
P_jejuni           cgtctgattagcttgttggcggggtaacggcccaccaaggcaacgatcagtaggggttct  277
P_veroralis        cgtctgattagcttgttggcggggtaacagcccaccaaggcgacgatcagtaggggttct  296
P_histicola        cgtctgattagcttgttggcggggtaacggcccaccaaggcaacgatcagtaggggttct  284
P_melaninogenica   cgtctgattagcttgttggcggggtaacggcccaccaaggcaacgatcagtaggggttct  296
P_oulorum          cgtctgattagcttgttggcggggtaacggcccaccaaggcaacgatcagtaggggttct  299
P_salivae          cgtctgattagcttgttggcggggtaacggcccaccaaggcaacgatcagtaggggttct  294
P_paludivivens     cgtctgattagcttgttggcggggtaacggcccaccaaggcaacgatcagtaggggttct  294
                   **************************** ************ ******************
                                                                 Universal F
P_copri            gagaggaaggtcccccacattggaactgagacacggtccaaactcctacgggaggcagca  356
P_jejuni           gagaggaaggtcccccacattggaactgagacacggtccaaactcctacgggaggcagca  337
P_veroralis        gagaggaaggtcccccacattggaactgagacacggtccaaactcctacgggaggcagca  356
P_histicola        gagaggaaggtcccccacattggaactgagacacggtccaaactcctacgggaggcagca  344
P_melaninogenica   gagaggaaggtcccccacattggaactgagacacggtccaaactcctacgggaggcagca  356
P_oulorum          gagaggaaggtcccccacattggaactgagacacggtccaaactcctacgggaggcagca  359
P_salivae          gagaggaaggtcccccacattggaactgagacacggtccaaactcctacgggaggcagca  354
P_paludivivens     gagaggaaggtcccccacattggaactgagacacggtccaaactcctacgggaggcagca  354
                   ************************************************************
```

```
                      ->
P_copri               gtgaggaatattggtcaatggrcgagagcctgaaccagccaagtagcgtgcaggatgacg  416
P_jejuni              gtgaggaatattggtcaatgggcgcgagcctgaaccagccaagtagcgtgcaggatgacg  397
P_veroralis           gtgaggaatattggtcaatgggcgagagcctgaaccagccaagtagcgtgcaggaagacg  416
P_histicola           gtgaggaatattggtcaatgggcgagagcctgaaccagccaagtagcgtgcaggatgacg  404
P_melaninogenica      gtgaggaatattggtcaatggacggaagtctgaaccagccaagtagcgtgcaggatgacg  416
P_oulorum             gtgaggaatattggtcaatgggcgagagcctgaaccagccaagtagcgtgcaggaagacg  419
P_salivae             gtgaggaatattggtcaatgggcgagagcctgaaccagccaagtagcgtgcaggatgacg  414
P_paludivivens        gtgaggaatattggtcaatgggcgaaagcctgaaccagccaagtagcgtgcaggatgacg  414
                      ********************* **  ** ************************** ****
                                                                       2R
                                                                       1R
P_copri               gccctatgggttgtaaactgcttttataagggaataaagtgagcctcgtgaggctttttg  476
P_jejuni              gccctatgggttgtaaactgcttttgtatggggataaagtcaatcacgtgtgattgtttg  457
P_veroralis           gccctatgggttgtaaactgcttttgtatggggataaagtcaatcacgtgtgattgtttg  476
P_histicola           gccctatgggttgtaaactgcttttgtatggggataaagtcagtcacgtgtgattgtttg  464
P_melaninogenica      gccctatgggttgtaaactgcttttgtatggggataaagttagggacgtgtccctatttg  476
P_oulorum             gccctatgggttgtaaactgcttttataggggaataaagtgatctacgtgtagtttattg  479
P_salivae             gccctatgggttgtaaactgcttttatgtggggataaagtgagctacgtgtagtttattg  474
P_paludivivens        gccctatgggttgtaaactgcttttatacagggataaagttagccacgtgtggctatttg  474
                      ************************* *   ** ******* *    ****    *  ***
                                                               Universal R
P_copri               catgtaccttatgaataaggaccggctaattccgtgccagcagccgcggtaatacggaag  536
P_jejuni              caggtaccatacgaataaggaccggctaattccgtgccagcagccgcggtaatacggaag  517
P_veroralis           caggtaccatacgaataaggaccggctaattccgtgccagcagccgcggtaatacggaag  536
P_histicola           caggtaccatacgaataaggaccggctaattccgtgccagcagccgcggtaatacggaag  524
P_melaninogenica      caggtaccatacgaataaggaccggctaattccgtgccagcagccgcggtaatacggaag  536
P_oulorum             tatgtaccctatgaataaggaccggctaattccgtgccagcagccgcggtaatacggaag  539
P_salivae             caggtaccacatgaataaggaccggctaattccgtgccagcagccgcggtaatacggaag  534
P_paludivivens        caggtactgtatgaataaggaccggctaattccgtgccagcagccgcggtaatacggaag  534
                       * ****   * ************************************************

P_copri               gtccgggcgttatccggatttattgggtttaaagggagcgtaggccggagattaagcgtg  596
P_jejuni              gtccgggcgttatccggatttattgggtttaaagggagcgtaggccggagattaagtgtg  577
P_veroralis           gttcgggcgttatccggatttattgggtttaaagggagcgtaggccggagattaagtgtg  596
P_histicola           gtccgggcgttatccggatttattgggtttaaagggagcgtaggctggagattaagtgtg  584
P_melaninogenica      gtccaggcgttatccggatttattgggtttaaagggagcgtaggctggagattaagtgtg  596
P_oulorum             gttcgggcgttatccggatttattgggtttaaagggagcgtaggccgtagattaagcgtg  599
P_salivae             gtccaggcgttatccggatttattgggtttaaagggagcgtaggccgtggattaagcgtg  594
P_paludivivens        gtccaggcgttatccggatttattgggtttaaagggagcgtaggccgtctgttaagcgtg  594
                      ** * **************************************** *    ***** ***
                                          3R                     Gray F
P_copri               ttgtgaaatgtagacgctcaacgtctgcactgcagcgcgaactggtttccttgagtacgc  656
P_jejuni              ttgtgaaatgtagacgctcaacgtctgacttgcagcgcatactggtttccttgagtacgc  637
P_veroralis           ttgtgaaatgtagacgctcaacgtctgacttgcagcgcatactggtttccttgagtacgc  656
P_histicola           ttgtgaaatgtagacgctcaacgtctgacttgcagcgcatactggtttccttgagtacgc  644
P_melaninogenica      ttgtgaaatgtagacgctcaacgtctgaattgcagcgcatactggtttccttgagtacgc  656
P_oulorum             ttgtgaaatgtagatgctcaacatctgacttgcagcgcgaactggtttacttgagtgtgc  659
P_salivae             ttgtgaaatgtagacgctcaacgtctgaattgcagcgcgaactggttcacttgagtatgc  654
P_paludivivens        ttgtgaaatgtagatgctcaacatctgaattgcagcgcgaactggcagacttgagtgtgc  654
                      ************** ******* ****   ********  *****    *******  **
                        Gray probe           Gray R
P_copri               acaaagtgggcggaattcgtggtgtagcggtgaaatgcttagatatcacgaagaactccg  716
P_jejuni              acaacgttggcggaattcgtcgtgtagcggtgaaatgcttagatatgacgaagaactccg  697
P_veroralis           acaacgttggcggaattcgtcgtgtagcggtgaaatgcttagatatgacgaagaactccg  716
P_histicola           acaacgttggcggaattcgtcgtgtagcggtgaaatgcttagatatgacgaagaactccg  704
P_melaninogenica      acaacgttggcggaattcgtcgtgtagcggtgaaatgcttagatatgacgaagaactccg  716
P_oulorum             acaacgtaggcggaattcgtcgtgtagcggtgaaatgcttagatatgacgaagaactccg  719
P_salivae             acaacgtaggcggaattcgtcgtgtagcggtgaaatgcttagatatgacgaagaactccg  714
P_paludivivens        gcaacgtaggcggaattcgtggtgtagcggtgaaatgcttagatatcacgaagaactcca  714
                       *** ** ************ ************************* ************

P_copri               attgcgaaggcagctcactggagcgcaactgacgctgaagctcgaaagtgcgggtatcga  776
P_jejuni              attgcgaaggcagctgacgggagcgcaactgacgcttaagctcgaaggtgcgggtatcga  757
P_veroralis           attgcgaaggcagctgacgggagcgccactgacgcttaagctcgaaggtgcgggtatcga  776
P_histicola           attgcgaaggcagctgacgggagcgcaactgacgctgaagctcgaaggtgcgggtatcga  764
P_melaninogenica      attgcgaaggcagctgacgggagcgcaactgacgcttaagctcgaaggtgcgggtatcaa  776
P_oulorum             attgcgaaggcagcttacgggagcacaactgacgctgaagctcgaaggtgcgggtatcaa  779
P_salivae             attgcgaaggcagcttacgggagcacaactgacgctgaagctcgaaggtgcgggtatcaa  774
P_paludivivens        attgcgaaggcagcttacgggagcacaactgacgctgaagctcgaaagtgcgggtatcga  774
                      *************** ** ***** * ********* ********* *********** *
```

Prevotella genus F →

```
P_copri            acaggattagataccctggtagtccgcacggtaaacgatggatgcccgctgttggtctga  836
P_jejuni           acaggattagataccctggtagtccgcacagtaaacgatggatgcccgctgttggtacct  817
P_veroralis        acaggattagataccctggtagtccgcacagtaaacgatggatgcccgctgttggtacct  836
P_histicola        acaggattagataccctggtagtccgcacagtaaacgatggatgcccgctgttggtacct  824
P_melaninogenica   acaggattagataccctggtagtccgcacagtaaacgatggatgcccgctgttggtacct  836
P_oulorum          acaggattagataccctggtagtccgcacagtaaacgctggatgcccgctgttggctcat  839
P_salivae          acaggattagataccctggtagtccgcacagtaaacgatggatgcccgttgttagttcac  834
P_paludivivens     acaggattagataccctggtagtccgcacggtaaacgatggatgcccgctgttgacccac  834
                   ***************************** ******* ********** ****

P_copri            --acaggtcagcggccaagcgaaagcattaagcatcccacctggggagtacgccggcaac  894
P_jejuni           ---ggtatcagcggctaagcgaaagcattaagcatcccacctggggagtacgccggcaac  874
P_veroralis        ---ggtatcagcggctaagcgaaagcattaagcatcccacctggggagtacgccggcaac  893
P_histicola        ---ggtatcagcggctaagcgaaagcattaagcatcccacctggggagtacgccggcaac  881
P_melaninogenica   ---ggtatcagcggctaagcgaaagcattaagcatcccacctggggagtacgccggcaac  893
P_oulorum          t--agagtcagcggccaagcgaaagcattaagcatcccacctggggagtacgccggcaac  897
P_salivae          ttatgaattagcgaccaagcgaaagcattaagcatcccacctggggagtacgccggcaac  894
P_paludivivens     c--tgggccagcggccaagcgaaagcattaagcatcccacctggggagtacgccggcaac  892
                            **** * ********************************************

P_copri            ggtgaaactcaaaggaattgacgggggcccgcacaagcggaggaacatgtggtttaattc  954
P_jejuni           ggtgaaactcaaaggaattgacgggggcccgcacaagcggaggaacatgtggtttaattc  934
P_veroralis        ggtgaaactcaaaggaattgacgggggcccgcacaagcggaggaacatgtggtttaattc  953
P_histicola        ggtgaaactcaaaggaattgacgggggcccgcacaagcggaggaacatgtggtttaattc  941
P_melaninogenica   ggtgaaactcaaaggaattgacgggggcccgcacaagcggaggaacatgtggtttaattc  953
P_oulorum          ggtgaaactcaaaggaattgacgggggcccgcacaagcggaggaacatgtggtttaattc  957
P_salivae          ggtgaaactcaaaggaattgacgggggcccgcacaagcggaggaacatgtggtttaattc  954
P_paludivivens     ggtgaaactcaaaggaattgacgggggcccgcacaagcggaggaacatgtggtttaattc  952
                   ************************************************************

P_copri            gatgatacgcgaggaaaccttacccgggcttgaattgcagaggaaggatttggagacaat  1014
P_jejuni           gatgatacgcgaggaa-ccttacccgggcttgaattgcagaggaaggatttagagataat  993
P_veroralis        gatgatacgcgaggaa-ccttacccgggcttgaattgcagaggaaggatttagagataat  1012
P_histicola        gatgatacgcgaggaa-ccttacccgggcttgaattgcagaggaaggatttagagataat  1000
P_melaninogenica   gatgatacgcgaggaa-ccttacccgggcttgaattgcagaggaaggatttagagataat  1012
P_oulorum          gatgatacgcgaggaa-ccttacccgggcttgaattgcagacgaacgattcagagatgaa  1016
P_salivae          gatgatacgcgaggaa-ccttacccgggcttgaattgcagacgaacgattcagagatgat  1013
P_paludivivens     gatgatacgcgaggaa-ccttacccgggcttgaattgcagaggaatttaccagagacggt  1011
                   **************** ************************ ***      ****

P_copri            gacgcccttcggggyctctgtgaaggtgctgcatggttgtcgtcagctcgtgccgtgagg  1074
P_jejuni           gacgcccttcggggcctctgtgaaggtgctgcatggttgtcgtcagctcgtgccgtgagg  1053
P_veroralis        gacgcccttcggggtctctgtgaaggtgctgcatggttgtcgtcagctcgtgccgtgagg  1072
P_histicola        gacgcccttcggggtctctgtgaaggtgctgcatggttgtcgtcagctcgtgccgtgagg  1060
P_melaninogenica   gacgcccttcggggtctctgtgaaggtgctgcatggttgtcgtcagctcgtgccgtgagg  1072
P_oulorum          gaggcccttcggggcgtctgtgaaggtgctgcatggttgtcgtcagctcgtgccgtgagg  1076
P_salivae          gaggcccttcggggcgtctgtgaaggtgctgcatggttgtcgtcagctcgtgccgtgagg  1073
P_paludivivens     aaagcccttcggggtctctgtgaaggtgctgcatggttgtcgtcagctcgtgccgtgagg  1071
                    * ***********  ********************************************

P_copri            tgtcggcttaagtgccataacgagcgcaacccctctccttagttgccatcaggtyaagct  1134
P_jejuni           tgtcggcttaagtgccataacgagcgcaacccctctcttcagttgccatcaggtcatgct  1113
P_veroralis        tgtcggcttaagtgccataacgagcgcaacccctctcttcagttgccatcaggtaatgct  1132
P_histicola        tgtcggcttaagtgccataacgagcgcaacccctgtctttagttgccatcaggttaagct  1120
P_melaninogenica   tgtcggcttaagtgccataacgagcgcaacccctctcttcagttgccatcaggttaagct  1132
P_oulorum          tgtcggcttaagtgccataacgagcgcaacccctttcttagttgccatcaggtcatgct  1136
P_salivae          tgtcggcttaagtgccataacgagcgcaacccctttttttcagttgccatcaggtgatgct  1133
P_paludivivens     tgtcggcttaagtgccataacgagcgcaacccctttctttagttgccatcaggtgatgct  1131
                   ********************************** *  * ************** * ***
```

```
P_copri             gggcactctggggacactgccaccgtaaggtgtgaggaaggtggggatgacgtcaaatca  1194
P_jejuni            gggcactctggagacactgccaccgtaaggtgtgaggaaggtggggatgacgtcaaatca  1173
P_veroralis         gggcactctggagacactgccaccgtaaggtgtgaggaaggtggggatgacgtcaaatca  1192
P_histicola         gggcactctggagatactgccaccgtaaggtgtgaggaaggtggggatgacgtcaaatca  1180
P_melaninogenica    gggcactctggagacactgccaccgtaaggtgtgaggaaggtggggatgacgtcaaatca  1192
P_oulorum           gggcactctgggaatactgccaccgtaaggtgtgaggaaggtggggatgacgtcaaatca  1196
P_salivae           gggcactctggagatactgccaccgtaaggtgtgaggaaggtggggatgacgtcaaatca  1193
P_paludivivens      gggcactctggagatactgccaccgtaaggtgtgaggaaggtggggatgacgtcaaatca  1191
                    ***********  * *********************************************

P_copri             gcacggcccttacgtccggggctacacacgtgttacaatggcaggtacagagagacggty  1254
P_jejuni            gcacggcccttacgtccggggctacacacgtgttacaatggccggtacagagggatggtg  1233
P_veroralis         gcacggcccttacgtccggggctacacacgtgttacaatggccggtacagagggacggtg  1252
P_histicola         gcacggcccttacgtccggggctacacacgtgttacaatggccggtacagagggacggtg  1240
P_melaninogenica    gcacggcccttacgtccggggctacacacgtgttacaatggccggtacagagggacggtg  1252
P_oulorum           gcacggcccttacgtccggggctacacacgtgttacaatggccggtacagagggcaggtt  1256
P_salivae           gcacggcccttacgtccggggctacacacgtgttacaatggccggtacagagcgttggtc  1253
P_paludivivens      gcacggcccttacgtccggggctacacacgtgttacaatggccggtacagagagacggtt  1251
                    ****************************************** ********* *  *** 
                                                                 <-- Prevotella genus R --
P_copri             scyygyaaagtsgatcaaatccttaaagcctgtctcagttcggactggggtctgcaaccc  1314
P_jejuni            taatgcaaattgcatcaaatcttgaaagccggtcccagttcggactggggtctgcaaccc  1293
P_veroralis         taatgcaaattgcatccaatcttgaaagccggtcccagttcggactggggtctgcaaccc  1312
P_histicola         taatgtaaattgcatccaatcttgaaagccggtcccagttcggactggggtctgcaaccc  1300
P_melaninogenica    taatgcaaattgcatccaatcttgaaagccggtcccagttcggactggggtctgcaaccc  1312
P_oulorum           gcatgtaaatgcgatcgaatcttaaaagccggtcccagttcggactgaggtctgcaaccc  1316
P_salivae           gtgtgcaaatacgatctaatccttaaagccggtcccagttcggactggggtctgcaaccc  1313
P_paludivivens      gcttgcaaaagtaatccaatccttaaagccggtcccagttcggactggggtctgcaaccc  1311
                        * ***    *** **** * ****** *** ************ ************
                    ---
P_copri             gaccccacgaagctggattcgctagtaatcgcgcatcagccatggcgcggtgaatacgtt  1374
P_jejuni            gaccccacgaagctggattcgctagtaatcgcgcatcagccatggcgcggtgaatacgtt  1353
P_veroralis         gaccccacgaagctggattcgctagtaatcgcgcatcagccatggcgcggtgaatacgtt  1372
P_histicola         gaccccacgaagctggattcgctagtaatcgcgcatcagccatggcgcggtgaatacgtt  1360
P_melaninogenica    gaccccacgaagctggattcgctagtaatcgcgcatcagccatggcgcggtgaatacgtt  1372
P_oulorum           gacctcacgaagctggattcgctagtaatcgcgcatcagccatggcgcggtgaatacgtt  1376
P_salivae           gaccccacgaagctggattcgctagtaatcgcgcatcagccatggcgcggtgaatacgtt  1373
P_paludivivens      gaccccacgaagctggattcgctagtaatcgcgcatcagccatggcgcggtgaatacgtt  1371
                    **** *******************************************************

P_copri             cccgggccttgtacacaccgcccgtcaagccatgaaagccgggggcgcctaaagtccgtg  1434
P_jejuni            cccgggccttgtacacaccgcccgtcaagccatgaaagccgggggtgcctgaagtccgtg  1413
P_veroralis         cccgggccttgtacacaccgcccgtcaagccatgaaagccgggggtgcctgaagtccgtg  1432
P_histicola         cccgggccttgtacacaccgcccgtcaagccatgaaagccgggggtgcctgaagtccgtg  1420
P_melaninogenica    cccgggccttgtacacaccgcccgtcaagccatgaaagccgggggtgcctgaagtccgtg  1432
P_oulorum           cccgggccttgtacacaccgcccgtcaagccatgaaagccgggggtgcctaaagtccgtt  1436
P_salivae           cccgggccttgtacacaccgcccgtcaagccatgaaagccgggggtgcctgaagtctgtg  1433
P_paludivivens      cccgggccttgtacacaccgcccgtcaagccatgaaagccgggggtgcctgaagtccgtg  1431
                    ********************************************* **** ***** ** 

P_copri             accgtaaggagcggcctagggcgaaactggtaattggggctaagtcgtaacaaggtaacc  1494
P_jejuni            accgcaaggatcggcctagggcaaaactggtgattggggctaagtcgtaacaaggtagcc  1473
P_veroralis         accggaaggatcggcctagggcaaaactggtgattggggctaagtcgtaacaaggtaacc  1492
P_histicola         accgcaaggatcggcctagggcaaaactggtgattggggctaagtcgtaacaaggtaacc  1480
P_melaninogenica    accgcaaggatcggcctagggcaaaactggtgattggggctaagtcgtaacaaggtaacc  1492
P_oulorum           accgcaaggatcggcctagggcaaaactggtaattggggctaagtcgtaacaaggtagcc  1496
P_salivae           accgcaaggaacggcctagggcaaaactggtgattggggctaagtcgtaacaaggtaacc  1493
P_paludivivens      accgcaaggaacggcctagggcaaaactggtaattggggctaagtcgtaacaaggtaacc  1491
                    **** ***** *********** ******** ************************* **

P_copri             --------------      1494
P_jejuni            gtaccggaagg---      1484
P_veroralis         --------------      1492
P_histicola         --------------      1480
P_melaninogenica    --------------      1492
P_oulorum           gtaccggaaggtgc      1510
P_salivae           --------------      1493
P_paludivivens      --------------      1491
```
